# The ideas about advanced life support and affecting factors at the end-stage of life in a hospital in Turkey

**DOI:** 10.1371/journal.pone.0181456

**Published:** 2017-07-21

**Authors:** Turgay Albayrak, İrfan Şencan, Ömer Akça, Esra Meltem Koç, Hilal Aksoy, Selim Ünsal, İskender Bülbül, Adem Bahadır, İsmail Kasım, Rabia Kahveci, Adem Özkara

**Affiliations:** 1 Yenimahalle Cigdemtepe Family Health Center, Ankara, Turkey; 2 Ankara Numune Training and Research Hospital, Department of Family Medicine, Ankara, Turkey; 3 Kayseri Training and Research Hospital, Department of Internal Medicine, Kayseri, Turkey; 4 Izmır Katip Celebi University Medical Faculty, Department of Family Medicine, Izmir, Turkey; 5 Pamukkale Pelitlibag Family Health Center, Denizli, Turkey; 6 Sefkat No 2 Family Health Center, Ankara, Turkey; 7 Guzelyurt State Hospital, Aksaray, Turkey; 8 Kalkandere No 1 Family Health Center, Rize, Turkey; 9 Corum Hitit University Medical Faculty, Department of Family Medicine, Corum, Turkey; Azienda Ospedaliero Universitaria Careggi, ITALY

## Abstract

**Background:**

The participation of the people in health decisions may be structured in various levels. One of these is participation in decisions for the treatment. “Advanced directives” is one of the examples for the participation in decisions for the treatment.

**Aim:**

We wanted to determine the decisions on advanced life support at the end-stage of life in case of a life-threatening illness for the people themselves and their first degree relatives and the factors effecting these decisions.

**Design and setting:**

The cross-sectional study was conducted with volunteers among patients and patient relatives who applied to all polyclinics of the Ankara Numune Training and Research Hospital except the emergency, oncology and psychiatry polyclinics between 15.12.2012 and 15.03.2013.

**Method:**

A questionnaire, the Hospital Anxiety Depression (HAD) scale, and Templer’s Death Anxiety Scale (TDA) were applied to all individuals. SPSS for Win. Ver. 17.0 and MS-Excel 2010 Starter software bundles were used for all statistical analysis and calculations.

**Results:**

The participants want both themselves and their first degree relatives included in end-stage decision-making process. Therefore, the patients and their families should be informed adequately during decision making process and quality communication must be provided.

**Conclusion:**

Participants who have given their end-stage decisions previously want to be treated according to these decisions. This desire can just be possible by advanced directives.When moral and material loads of end-stage process are taken into consideration, countries, in which advanced directives are practiced, should be examined well and participants’ desire should be evaluated in terms of practicability.

## Introduction

How much and how should the public be involved in the structure of health care? The participatory role of the public can be imagined as a ladder, and in literature, the step that public involvement should occur is widely discussed [1]. Public participation in health decisions can occur at many levels. One of these is participation in treatment decisions. "Advance directives" are an example of participation in treatment decisions. Advance directives are written documents that state the patients' healthcare wishes in case of severe illness or identify the person that will make decisions for the patient should he/she become incapacitated to make his/her own decisions.

The most common two advance directives used in the United States of America are "living wills" and "health care power of attorney". A living will is a document that states what type of medical treatment you desire at the terminal period of your life. For example, a patient may state whether he/she wishes to be connected to a mechanical ventilator or express the conditions in which he/she wishes to receive mechanical ventilator support. Health care power of attorney is a legal document; dated and signed in the presence of a witness that is proof that the patient has authorized a certain person to make health decisions on her/his behalf should she/he become incapacitated. Directives about the treatments permitted and prohibited by the patient can also be added to this document. [2, 3] Living wills state how much life support the patient wishes to receive when she/he is too sick to express her/his own decisions. Living wills communicate the patients' wishes to her/his family and doctor and ensure that the patient receives the treatment he/she requested [4]. Appointing a health power of attorney is a more flexible approach than life wills and may also covers unexpected conditions. The health power of attorney has no authority to make business-related or financial decisions and can only make decisions about health conditions. Appointing a health power of attorney allows those delivering health care to make decisions such as ending or withdrawing life support treatment if the patient is in terminal period, a coma, has severe dementia or is in a continuous vegetative state [5].

Within the healthcare system of our country, it is seen that most patients do not actively participate in health decisions. Conventionally, medical decisions are made by doctors. There is a regulation on “Patients’ Rights” in Turkey. In this regulation it is stated that in a life threatinig situation or in an unconscious state when the consent of patient can not be taken, medical intervation is not under consent of anybody [6]. The closest directive to advance directive is defined in the 14. Item of The Laon Organ Transplantation. This law permits donation of organ on donor’s own initiative[7]. Medical decisions can be defined as a "power" that is generated during the delivery of healthcare. Sharing this power increases the satisfaction of the recipient and provider of healthcare, the functionality of the services, and compliance with the treatment. However, the number of studies conducted in our country about "making joint decisions" is insufficient.

With this study our objective is to:

1. Determine the decisions made by people about intensive care and life support when they or their first-degree relatives face a potentially life-threatening disease and the factors that affect these decisions,
2. Determine the effect of family physicians on these decisions that are very important for the patients,
3. Identify the level of individuals' participation in these decisions and whether the target of effective health services and functions which is the public wishes to take a more active role.

## Material and method

The ethical approval for the study was taken from Ethics Committee of Ankara Numune Training and Research Hospital. The number of ethical approval is 2012–487.

The cross-sectional study was conducted with volunteers among patients and patient relatives that applied to all polyclinics of the Ankara Numune Training and Research hospital except the emergency, oncology and psychiatry polyclinics between 15.12.2012 and 15.03.2013. Individuals under 20 years of age, over 65 years old, those diagnosed with psychosis, healthcare workers and those with any life-threatening disease were excluded from the study.

### Collection of data

A questionnaire consisting of 37 questions prepared by the researchers, the Hospital Anxiety Depression (HAD) scale, and Templer’s Death Anxiety Scale (TDA) were applied to all individuals that participated in the study. Survey was conducted face to face in the hospital and it took about 20–30 minutes for each participant.

#### Data collection instruments

The questionnaire contains questions prepared to determine the socio-demographic characteristics, features of the personal and family history, the advanced life support decisions and the factors that may affect these decisions, and the role of family physicians in this process. Literature and the clinical experience of the researchers were used to prepare the questions about advanced life support decisions and the factors that affect these decisions.

#### The hospital anxiety depression scale

The HAD is a 14-item self-report scale commonly used in hospital settings to determine the risk for anxiety and depression, to measure the level and changes in the severity of depressive symptoms in patients. The cut-off point for the subscales of the HAD is ≥ 8 [8]. The HAD was translated to Turkish by Aydemir and colleagues [9].

#### Templer’s death anxiety scale

Templer’s DAS was developed by Templer in 1970. This scale is a 15-item true/false survey that measures the anxiety and fear of a patient of his/her own death or death risk. The total score may be between 0 to 15. High scores reflect death anxiety. The DAS has been translated into Arabic and Spanish and the reliability and validity study in Turkey has been conducted by Senol in 1989 [10,11]. The first 9 items of the scale are given the value "1" for the answer yes and "0" for the answer no, the remaining 6 items are given the value "0" for the answer yes and "1" for the answer no. The lowest score of the scale is 0 and the highest is 15. Scores between 0 to 4 reflect mild, scores between 5 to 9 reflect moderate, scores between 10 to 14 reflect severe death anxiety and 15 points reflect death anxiety at a panic level. The "test-retest" reliability of the scale is 0.83 [10–12].

#### Pilot study

After the survey questions were prepared the survey was applied to 20 people for the pilot study. After evaluating the data gathered from the pilot study, the questions that appeared to be misunderstood were re-written.

### Evaluation of the data and statistical analysis

The normality of the distribution of continuous variables was assessed graphically using the Shapiro-Wilk test. Descriptive statistics were expressed as mean ± standard deviation (SD). Categorical and classified variables were expressed using numbers, percentages, and crosstabs. When continuous variables were compared with regard to experiment groups the student's t-test or the Mann-Whitney test was used depending on normal distribution. The differences between the categorical variables inter se were assessed using chi-square, chi-square likelihood ratio or Fisher's exact chi-square by preparing crosstabs. SPSS for Win. Ver. 17.0 (SPSS Inc. Chicago, IL., USA) and MS-Excel 2010 Starter software bundles were used for all statistical analysis and calculations. P < 0.05 was regarded as the sign of significant differences while assessing the results of statistical analyses.

### Findings

The data of 340 people was used in the study. Among the participants 53.8% (n = 183) were male and 46.2% (n = 157) were women. The socio-demographic data of the participants is presented in Table 1.

**Table 1:** Sociodemographic data.

		n	%
**Gender**	**female**	**157**	**46,2**
	**male**	**183**	**53,8**
**Age**	**18–29 years**	**100**	**29,4**
	**30–39 years**	**135**	**39,7**
	**40–49 years**	**62**	**18,2**
	**50–65 years**	**43**	**12,7**
**Marital status**	**married**	**232**	**68,2**
	**single**	**95**	**27,9**
	**widow**	**7**	**2,1**
	**divorced**	**6**	**1,8**
**Number of children**	**0**	**123**	**36,2**
	**1**	**65**	**19,1**
	**2**	**100**	**29,4**
	**3**	**35**	**10,3**
	**4 ve üzeri**	**18**	**5,3**
**Education level**	**illiterate**	**1**	**0,3**
	**literate**	**4**	**1,2**
	**primary school**	**37**	**10,9**
	**secondary school**	**28**	**8,2**
	**high school**	**94**	**27,6**
	**university**	**176**	**51,8**
**Living place**	**Village**	**19**	**5,4**
	**Town**	**91**	**26,8**
	**City center**	**230**	**67,6**
**Job**	**not working**	**31**	**9,1**
	**regular job**	**210**	**61,8**
	**Irregular job**	**11**	**3,2**
	**own job**	**13**	**3,8**
	**retired**	**21**	**6,2**
	**house wife**	**54**	**15,9**
**Total income (Euro)**	**0–191**	**32**	**9,4**
	**192–244**	**44**	**12,9**
	**245–794**	**164**	**48,2**
	**795 and over**	**100**	**29,5**

While 75.3% (n = 256) of the participants had no chronic disease, 24.7% (n = 84) had more than one chronic disease, and while 20% (n = 68) of the participants had a medication they regularly used, 80% (n = 272) had no regular medications. Only 10% (n = 34) of the patients stated that they received treatment for a psychiatric disease.

It was identified that 34.1% (n = 116) of the patients in our study had a history of surgery, 17.1% (n = 58) had previously faced a life-threatening condition, and 33.8% (n = 115) had a history of inpatient treatment in hospital.

Among those that completed the questionnaire, 81.2% (n = 276) expressed that they had a relative that was treated as an inpatient in hospital before, 42.1% (n = 143) said that they had a bedridden relative and/or acquaintance, and 67.4% (n = 229) had previously met a patient that was about to die.

67.1% of the participants expressed they would want interventions such as cardiac massage and mechanical ventilation to be performed in patients with an incurable disease even during the terminal period. In face of the same condition, 55% of the participants requested interventions to be performed. A higher ratio of participants (82.1%) requested interventions for their relatives.

There were statistically significant associations between the situations below:

Between interventions being requested in the terminal period by participants for themselves and the area of residence of the participant (p = 0.004).

Between participants requesting interventions to be performed for themselves and the effect of these interventions on the survival and life comfort of the patient (p<0.0001).

Between participants requesting interventions for themselves in their terminal period and their surgical history (p = 0.044). Individuals among the participants with a history of surgery request interventions to be performed in their terminal period more than those with no surgical history when they are diagnosed with an incurable disease.

Between participants requesting interventions to be performed for their first-degree relatives and the presence of chronic diseases in the participants (p = 0.017).

Between requests to perform interventions for a person in the terminal period of an incurable disease and requests to be informed about their condition if they develop a serious disease in the future (p = 0.03).

Between the effects of interventions that would be performed in the terminal period on the survival and life comfort of the patient and the participant having a relative that was treated in hospital (p = 0.006) and having previously met a near death patient (p = 0.04).

Among the 340 participants 75% (n = 225) had a plan for the future, 10.9% (n = 37) did not have a plan for the future and 14.1% (n = 48) stated that they had never given this any thought before.

Of the participants, 12.1% (n = 41) expressed they had considered "desire to die" before and 87.6% (n = 298) had no such thoughts. One person did not answer this question.

The most common reasons expressed by individuals with prior desire to die were familial reasons, illness, and financial reasons in that order of frequency. Those without desire to die gave the explanation that it was against religious beliefs and that it was not a plan for the future.

Among the participants, 45% want their first-degree relative to be informed about their condition if they develop a life-threatening disease in the future. Even if their first-degree relatives are in the terminal period of their lives, 82.1% of the participants request that interventions be performed. 48.8% of the participants believe that it would not be appropriate to remove their relatives that are in a vegetative state in the intensive care unit from the mechanical ventilator. The participants want to consult their families (58.8%) and the doctor following their patient (30%) the most about decisions concerning their first-degree relatives.

### The HAD scale results

#### HAD anxiety (HAD A)

The anxiety scores of the participants in our study were over 10 points in 19.7% (n = 67) and equal or under 10 in 80.3% (n = 273).

#### HAD depression (HAD D)

The depression scores of the participants in the study were over 7 in 31.5% (n = 107) and under 7 in 68.5% (n = 233).

According to this, by reviewing the anxiety and depression scores of the participants the average anxiety score was identified as 7.60 ± 3.721 (Min: 0, Max: 20), and the average depression score was 5.99 ± 3.522 (Min: 0, max: 20). Among the patients, 20% scored over the threshold in the anxiety subscale, and 31% scored over the threshold in the depression subscale. In other words, 20% of the participants are at risk for anxiety and 31% are at risk for depression.

#### Templer's death anxiety scale results

Among the participants 17.7% (n = 60) had mild, 57.7% (n = 196) had moderate, 24.3% (n = 83) had severe and 0.3% (n = 1) had panic level death anxiety.

## Discussion

The terminal period is the last stage of a persons’ life before death. Being in the terminal period may be associated with an acute or chronic disease or directly with age. Whatever the reason, the reality of the terminal period is that the person's death is expected within weeks or months and that this cannot be prevented by medical interventions either [13]. Both the physical and psychosocial needs of patients must be met during the terminal period. For this reason some patients spend their terminal period in hospital intensive care units for their physical needs to be met.

Patients with multi-organ damage or severe organ damage in intensive care units are kept alive by means of intensive and expensive advanced treatments. Despite this, deaths in ICUs are frequent [14].

Although medical interventions or treatments that would be applied in the ICU or any other setting do not significantly affect the outcome, the participants of our study believed otherwise and answered the questions in the survey accordingly.

We wonder which factors affect the decisions patients with incurable diseases make about interventions being or not being performed during the terminal period of their lives. Perhaps this question, the focus of our study and one of the questions we wanted to find the answer to most, was our greatest motivator while conducting this study and guided our way of thinking.

As the participants’ area of residence gradually moved from city centers to districts, towns, and villages they increasingly want interventions to be performed during the terminal period of their lives if they are diagnosed with an incurable disease. The most important reasons are increased awareness and knowledge about the issue and the increased stress with the complexity and the difficulty of living conditions moving from villages towards city centers.

The most frequent surgeries identified in the history of our patients were operations such as appendectomies, thyroidectomies, inguinal herniorrhaphies, pilonidal sinus surgeries, and cesarean sections. These surgical interventions performed are relatively major surgeries that do not cause problems, and most of these patients had achieved cure after these procedures. Therefore, we believe that participants with a history of surgery requested interventions to be performed in their terminal period because they assumed that they would benefit from these interventions in the same manner as they did from their prior surgeries.

Participants request interventions both for themselves and for individuals in the terminal period. We were pleased with the fact that the participants considered and wished the same as they would for themselves in face of such an important condition for any other person in the society as this shows that our society's moral values have not been corrupted.

Participants request what they think is the correct decision for themselves more strongly for their first-degree relatives. Besides this, the participants request interventions for themselves and prefer that a family member makes decisions on their behalf if needed. This result is extremely important for guiding about the stakeholders of the joint end-of-life decision-making process.

Participants request interventions to be performed for themselves because they believe that these interventions will positively affect the survival and life comfort of the patients. Actually, interventions that are performed in such patients have no positive effect on the survival and life comfort of the patient. We believe that this belief of the participants is due to the lack of patient knowledge about terminal care and the medical interventions that will be performed, and also because they have a more sentimental approach to the situation. In a study conducted in Spain 135 patients’ information was used to determine the frequency of intensive care planning. Intensive care planning was found in16.3% (n:22) of patients. Only 2 of them had a living will and these patients did not want any intervention whwn the risk of death was high [15].

There was a statistically significant association between participants requesting interventions to be performed for themselves and interventions being performed in accordance with the decision patients would make beforehand. According to this, those that did not request interventions to be performed in their terminal period do not want the decision they made in advance to be executed. This is because patients do not feel their knowledge is sufficient to make a decision, nor do they have confidence in their knowledge to make these decisions in face of such an important event.

While we were planning this study we reviewed the joint decision-making process for terminal care decisions and various approaches used around the world and we discovered the concept of "health power of attorney.” According to this, a person authorized in advance via a dated, witnessed official document to make health decisions on behalf of an individual unable to make decisions due to any reason can make decisions and participate in the joint decision-making process. Besides this, in current medical practice consent is obtained from the patient's family or relatives before certain medical procedures in Turkey and also many other countries. After realizing this, determining what terminal care decisions our participants would make on behalf of their first-degree relatives and identifying the factors that influence these decisions became another important goal.

More participants that do not suffer from any chronic diseases request interventions to be performed for their relatives in the terminal period. We identified that an important factor that led participants that did not suffer from chronic diseases to make these decisions is the fact that they have not experienced the course of chronic diseases or the consequent lifestyle changes.

The individuals among our participants that had not met a near-death person request interventions to be performed for their first-degree relatives. In patients that enter the terminal period because of an acute or chronic disease, this period is rather stressful for both the patient and the patient's family. People that witness this period, request non-intervention while making terminal care decisions regardless of who the subject is. Some believe that such interventions are painful for the patient and the patient's relatives.

In our study it can be understood that the participants want to be informed of their condition if they develop a life-threatening disease and also request interventions to be performed for patients in the terminal period.

Participants also request interventions for a person in the terminal period with an incurable disease. However, the focus of attention here should be the answers given to the question on “being informed about a serious disease that may develop in the future” on behalf of themselves and their first-degree relatives” which was found to be statistically significant with this question. The answers participants gave about themselves show that they want to be informed about such conditions. However, the answers they gave on behalf of their relatives show that they are unsure whether their first-degree relatives should be informed about such conditions. When we examine this condition further, we got some answers from the participants about the reasoning behind this. In accordance with the answers we obtained, we gathered the anxiety and fear of the participants related to this subject under two groups. Firstly, in the face of such news, they fear that their relatives will become extremely upset and lose their zest for life. Secondly, the participants fear that the emotional condition their relatives will experience in response to this news will negatively affect the treatment of their disease.

For a patient in the terminal period, death is expected within weeks or months, and this cannot be prevented by medical interventions either [13]. This is a medical fact. Knowing this will obviously influence the decisions that will be made by patients during this period. Besides this, how well is this medical fact understood by the public that is the receiving end of care within the health system? With this purpose, we asked the question "how do terminal care interventions affect the survival and life comfort of patients?" and attempted to identify the factors that influence the answers given to this question.

The participants that did not have any relatives that had been treated as an in-hospital patient before and those that had not met a near-death patient believe that interventions that will be performed positively affect the survival and life comfort of patients.

By reversing our point of view based on our dialogues with the participants, we can explain the two results obtained. Patient visits are very important for the formation of a certain health culture. A person's health culture is affected by the conversations they have with her/his own patient, other patients and patient relatives, observations they make, and situations they witness (the cries of people that lost a relative, seeing intubated patients or amputees in the corridor, etc.) during hospital visits to a relative or an acquaintance in hospital. It is for this reason that the participants that have a relative that had been treated in hospital and participants that had previously met a near-death patient do not believe that terminal period interventions positively affect the survival and life comfort of patients.

The participants that have plans and expectations about the future believe that terminal period interventions have a positive effect on the survival and life comfort of the patient.

Participants want their families to make terminal care decisions on their behalf and believe that such interventions would positively affect the survival and life comfort of the patient. Family relationships are very important during intensive care planning. In one study, it was stated that parents with problem parent-child relationships had fewer chances of completing intensive care planning [16].

In a study conducted in 37 intensive care units of 17 European countries the rates of interviews with families are 84% in North European countries, 66% in Middle European countries and 47% in South European countries [17]. When we look at the countries in the other regions of the world, the participation of the families in recent decisions is largely different. For example, family participation in recent decisions has been reported 100% in India, 98% in Hong Kong, 79% in Lebanon, 72% in Spain and 44% in France [18–22].

While we were planning our study, we predicted that the answers that would be given to the questions we prepared in accordance with the topic and the purpose of our study might be influenced by the general anxious or depressed mood of the patients. In line with our thought, we used the HAD scale to determine whether the results of our study differed according to the general anxiety and depression of the patients.

According to the results, the anxiety scores were higher in patients that previously had a desire to commit suicide, and the depression scores were higher in those with chronic diseases. In previous studies it was determined that age, gender, personality traits, socio-cultural factors, developmental process, religious beliefs, and fatal disease states were most situations associated with death anxiety [23–26]. There is no consensus on how age change affects death anxiety. However, in the majority of studies, it is reported that in the elderly, the anxiety of death is lower than in adolescents and young adults [25].

In some studies death anxiety was found higher in women than men, but in some studies significant difference was not found [26–27]. Men are afraid of extinction of lineage and not living more, women are afraid of not leaving enough life conditions for their family [25].

In most of the studies conducted in Turkey death anxiety was found higher in married people than single people. But in some studies no relationship was found [28–30].

The depression score was lower in the participants that wished that they and their relatives be informed about a serious disease.

Participants with low depression scores want such important conditions to be told to them and their first-degree relatives. On the other hand, participants with high depression scores requested that they or their relatives not be informed about such important conditions. Participants with high depression scores predict that their current depressive condition will be affected negatively if they are informed about such events. In addition, the current depressive mood of patients forces them to make somewhat defensive decisions at the very beginning of the event, at the stage of being informed about the presence of a disease because their motivation will be negatively affected during the treatment period after the diagnosis of such a condition if they are aware of the situation. This result is very important as it supports our prediction that a depressive mood might influence the answers.

According to the results of the HAD-D we applied to the participants, there was a statistically significant association between the participant writing the decisions they make in advance about terminal period interventions in the form of a document resembling a will and the participants willing to give this to their family physicians (p = 0.009). The depression scores were lower in the participants that wanted to write their decisions on a document resembling a will and to give it to their family physician than they were in the participants that did not want to do this.

Participants with low depression scores want to make advance decisions about terminal period interventions and to give these decisions to their family physician in written form like a will. On the other hand, participants with high depression scores do not want to make decisions about terminal care interventions in advance nor do they want to give these decisions to their family physician in written form like a will. Depressive mood affects the decisions participants make about such an important subject. Having a high depression score prevents the patient from making decisions and completing the important step of writing a will and giving it to their family physician which facilitates the execution of the decision. On the other hand, low depression scores enable the participants to make decisions easily and boldly about such an important subject that is not even a topic of discussion and is far from being practiced (terminal care decisions) in our country. This result is very important as it supports the prediction we had while we were planning our study that depressive moods might influence the answers given.

According to the results obtained from the Templer DAS in our study, there was a statistically significant association between death anxiety and some of the variables and questions in the questionnaire.

While anxiety was more intense in women than it was in men in some studies conducted on death anxiety, others did not detect a significant association [15–16]. The statistically significant association between death anxiety and the gender variable (p<0.0001) revealed that death anxiety was more common in the female participants of the questionnaire than it was in the males. Both while we were applying the questionnaire and after it was applied, we observed that the most important reason of death anxiety in women and in particular women with children was the women’s' concern about their children’s futures.

Death anxiety and the presence of illness often concur. In other words, some phases of diseases cause death anxiety to develop and intensify [31]. In the study we conducted, participants having a bedridden relative or acquaintance increases the death anxiety of the participants (p = 0.04). This is important as it shows that people are unable to isolate themselves from such illnesses in their environment and that they have dynamic interactions with their environment.

There was a statistically significant association between the death anxiety of the participants and where they want to be when they are near death. According to this, death anxiety is higher in participants that want to be in their own homes when they are near death than it is in those that want to be in the hospital (p = 0.022). As a result of the conversations we had while performing the questionnaire, we concluded that a large portion of the participants (85.29%) wish to be in their own homes beside their loved ones during the last period of their lives. However, we observed that they are concerned that the condition they are in while they are living the last days of their lives will upset their loved ones, and this concern leads to the development of death anxiety. There was less death anxiety in the participants that wanted to spend the last period of their lives in the hospital due to reasons such as the confidence given by the availability of continuous medical care and the means to perform the needed interventions promptly.

We assessed the association of death anxiety with the current depressive or anxious mood of the participants. While there was a positive association between the general anxiety level and death anxiety [32], in our study there was a significant association with depression instead of anxiety (p<0.0001). According to this, death anxiety increases as the HAD-D score increases.

While the health services provided are assessed quantitatively and qualitatively, feedback from the receiving end of the services and particularly the patients is also rather important. The health system and health services provided can be reviewed according to this feedback and modified in accordance with current means and medical facts. In other words, this feedback somewhat plays the role of a guide. The satisfaction of the receiver of these services is probably the most important aspect of this feedback.

Patient satisfaction is the difference between the levels of health services expected and perceived by patients [33]. Patient satisfaction provides information about the level of satisfaction of the patient's values and expectations during the processes of receiving health services. The main authority of patient satisfaction is the patient. Patient satisfaction is the fundamental measure that reflects the quality of the care delivered [34]. On the other hand, because the cost of health services is gradually increasing, patient satisfaction is becoming evidence required to make decisions that use current resources adequately and effectively (19) [35].

By means of advancing technology and increasing opportunities, the health services providers have become advantageous in managing diseases. While providing health services, the pathological condition described as the ailment by the patient is identified using current knowledge and means, and it is treated by medical interventions and treatments. As much as the positive (being treatable) or negative (incurable) outcomes of the medical interventions and treatments are important, the clinical method applied to the patients and their relatives during these processes are also important. The method that should be applied is the method of patient-centered care [36]. By this means, the satisfaction of the receivers and providers of health care, the functionality of the services provided and treatment compliance increases, and conflicts that occur while delivering these services decrease [37]. By means of this method, ordering excessive tests and repeat applications of patients are prevented; this contributes to the health care system [36].

We believe that the answers and feedback we received during this study and the interest of the patients and their relatives in this subject which is the heart of the patient-centered clinical method are rather important as they show that this clinical method is applicable. How much do patients and their relatives want to participate in medical decisions? What answers do they give about medical events? What influences these decisions made? In this respect this study is important. However, medical decisions have a very wide scope. By focusing only on terminal period decisions in this study, we specialized on this topic. Besides this, terminal period decisions are a very strategic subject because terminal care decisions are very important and serious decisions to the participants. Identifying the participation status of the participants through making decisions on such an important subject is important to predict their participation in other medical diseases. In other words, the participants' favor towards this study should be assessed well as guidance to facilitate the participation of patients and their relatives in medical decision-making processes. Through the opinion that develops by this means, the applicability of the patient-centered method can be assessed, and strategies can be developed in this direction. One other feature that makes terminal period decisions a strategic topic is the fact that it is not discussed or practiced in our country. The fact that there are various practices and discussions about this subject in other countries makes this subject more important and interesting.

## Supporting information

S1 File Questionnaire. (DOCX)

## References

[1] Kahveci R. A step towards democratization of health care services: patient involvement in terminal life support decisions. Turkiye Klinikleri J Med Ethics. 2007;15: 90–93.

[2] Advance directives: Be prepared, in people's medical society. Newsletter. 1992;11: 4–5.

[3] Park DC, Eaton TA, Larson EJ, Palmer HT. Implementation and impact of the patient self-determination act. South Med J.1994;87(10): 971–977. 7939924 10.1097/00007611-199410000-00002

[4] Ellentuck AB. A will is a start: Here's what else you need. Nation's Business. 1992;80: 79–80.

[5] Sypher B. Initiating discussions about advance directives: the family physician's role. Am Fam Physician. 2002;65(12): 2443–2444. 12086236

[6] Hasta Hakları Yönermeliği. Resmi Gazete Tarihi: 01.08.1998 Resmi Gazete Sayısı: 23420 (Regulation on Patints’ Safety. Official paper date:01.08.1998 Official paper number:23420.)

[7] Guven T. TURKEY, in Country Reports on Advance Dırectives. Institute of Biomedical Ethics, University of Zurich: Zurich, Switzerland 18–22 6 2008.

[8] Zigmond AS, Snaith RP. The hospital anxiety and depression scale. Acta Psychiatr Scand. 1983;67(6): 361–370. 6880820 10.1111/j.1600-0447.1983.tb09716.x

[9] Aydemir O, Guvenir T, Kuey L. Validity and reliability of Turkish version of hospital anxiety and depression scale. Turkish Journal of Psychiatry. 1997;8:280–287.

[10] Abdel-Khalek AM. Kuwait anxiety and death anxiety in Egyptian and Spanish nursing students death studies. College of Social Sciences, Kuwait University. 2005; 29:157–169. 10.1080/07481180590906174 15822243

[11] Senol C. Death-related anxiety and fear in the elderly in living facilities in Ankara. Postgraduate thesis, Social Sciences Institutes Ankara University, Ankara. 1989.

[12] Akça FK, Kose A. Adaptation of the death anxiety scale: Validity and Reliability Study. Clinical Psychiatry. 2008;11: -16.

[13] Karan MA, Akın S. Approach to the elderly terminal patient. Klinik Gelişim Dergisi.2012; 25: 90–94.

[14] Curtis JR, Vincent JL, Ethics and end-of-life care for adults in the intensive care unit. Lancet. 2010;376(9749): 1347–1353. doi: 10.1016/S0140-6736(10)60143-2 20934213 10.1016/S0140-6736(10)60143-2

[15] Diestre Ortin G, González Sequero V, Collell Domènech N, Pérez López F, Hernando Robles P. Advance care planning and severe chronic diseases. Rev Esp Geriatr Gerontol,. 2013;48(5):228–231. doi: 10.1016/j.regg.2013.01.001 23643615 10.1016/j.regg.2013.01.001

[16] Carr D, Moorman SM, Boerner K. End-of-life planning in a family context: does relationship quality affect whether (and with whom) older adults plan? J Gerontol B Psychol Sci Soc Sci.2013;68(4): 586–592. doi: 10.1093/geronb/gbt034 23689997 10.1093/geronb/gbt034 PMC3674735

[17] Sprung CL, Cohen SL, Sjokvist P, Baras M, Bulow HH, Hovilehto S, et al End-of-life practices in European intensive care units: the Ethicus Study. JAMA. 2003;290(6): 790–797. doi: 10.1001/jama.290.6.790 12915432 10.1001/jama.290.6.790

[18] Yazigi A, Riachi M, Dabbar G. Withholding and withdrawal of life-sustaining treatment in a Lebanese intensive care unit: a prospective observational study. Intensive Care Med. 2005; 31(4): 562–567. doi: 10.1007/s00134-005-2578-4 15750799 10.1007/s00134-005-2578-4

[19] Mani RK, Mandal AK, Bal S, Javeri Y, Kumar R, Nama DK, et al End-of-life decisions in an Indian intensive care unit. Intensive Care Med. 2009;35(10): 1713–1719. doi: 10.1007/s00134-009-1561-x 19568731 10.1007/s00134-009-1561-x

[20] Buckley TA, Joynt GM, Tan PY, Cheng CA, Yap FH. Limitation of life support: frequency and practice in a Hong Kong intensive care unit. Crit Care Med. 2004;32(2):415–420. doi: 10.1097/01.CCM.0000110675.34569.A9 14758157 10.1097/01.CCM.0000110675.34569.A9

[21] Esteban A, Gordo F, Solsona L, Alia I, Caballero J, Bouzo C, et al Withdrawing and withholding life support in the intensive care unit: a Spanish prospective multi-centre observational study. Intensive Care Med. 2001;27(11): 1744–1749. doi: 10.1007/s00134-001-1111-7 11810117 10.1007/s00134-001-1111-7

[22] Ferrand E, Robert R, Ingrad P, Lemaire F. Withholding and withdrawal of life support in intensive-care units in France: a prospective survey. French LATAREA Group. Lancet. 2001; 357(9249): 9–14. 11197395 10.1016/s0140-6736(00)03564-9

[23] Pollack J. Correlates of death anxiety: a review of empirical studies. Omega: Journal of Death and Dying. 1980; 10(2): 97–121.

[24] Aday RH. Belief in afterlife and death anxiety: correlates and comparisons. Journal of Death and Dying. 1985; 15(1):67–75.

[25] Kastenbaum R. Death anxiety,Tempe AZ,USA Arizona State University 2007.

[26] Conte HR, Weiner MB, Plutchik R. Measuring death anxiety: conceptual, psychometric, and factor-analytic aspects. J Pers Soc Psychol. 1982;43(4):775–785. 7175677 10.1037//0022-3514.43.4.775

[27] Keller JW, Sherry D, Piotrowski C. Perspectives on death: a developmental study. J Psychol. 1984;116(1st Half): 137–142. doi: 10.1080/00223980.1984.9923628 6699791 10.1080/00223980.1984.9923628

[28] Erdogdu MY, Özkan M. Farklı dini inanışlardaki bireylerin ölüm kaygıları İle ruhsal belirtiler ve sosyo-demografik değişkenler arasındaki ilişkiler. İnönü Üniversitesi Tıp Fakültesi Dergisi.2007; 14(3):171–179.

[29] Yıldız M. Dini hayat İle ölüm kaygısı arasındaki İlişki üzerine bir araştırma, Doctoral thesis, Social Sciences Institutes, Dokuz Eylül University:İzmir.1998.

[30] Turgay M. Ölüm korkusu ve kişilik yapısı arasındaki ilişki, Doctoral thesis,Social Sciences Institutes, Istanbul University: Istanbul. 2003.

[31] Kastenbaum R: Death anxiety In Encyclopedia of Stress (Second Edition), Tempe AZ, USA Arizona StateUniversity; 2007pp:717–722. https://doi.org/10.1016/B978-012373947-6.00113-6

[32] Ozturk Kalaoglu Z. Death anxiety in elderly individuals, Thesis. Department of Psychiatry, Cukurova University: Adana.2010.

[33] Ozer A, Cakıl E. Factors that affect patient satisfaction–a review. Journal of Medical Research. 2007;5 (3):140–143.

[34] Carr-Hill RA. The measurement of patient satisfaction. J Public Health Med. 1992;14(3):236–249. 1419201

[35] Williams B. Patient satisfaction: a valid concept? Soc Sci Med. 1994;38(4):509–516. 8184314 10.1016/0277-9536(94)90247-x

[36] Mc Whinney IR, Freeman T.: Clinical Method In Mc Whinney Family Medicine, Turkish, First Edition Istanbul: Medical Academy 2012pp:140–192.

[37] Stewart M, Brown JB, Weston W, McWhinney IR, McWilliam C, Freeman TR. Patient-centered medicine: transforming the clinical method Thousand Oaks, CA: Sage Publications; 1995.

