# Supplementary material for: The ideas about advanced life support and affecting factors at the end-stage of life in a hospital in Turkey
Source: PLoS One. 2017 Jul 21;12(7):e0181456. doi: 10.1371/journal.pone.0181456 (PMC5521791; doi:10.1371/journal.pone.0181456)
Supplement: S1 File — (DOCX) [file pone.0181456.s001.docx]

**Questionnaire**

**1-Gender**

a)Male b)Female

**2- Age:**

**3-Marital status:**

a)Single b)Married c)Widowed d)Divorced

**4-Number of children:**

**5- Education level:**

1. Illiterate b)Literate c)Primary school d)Secondary school e)High school f)University

**6-Where do you live?**

a)Village b)Town c)District d)City center

**7-Occupation:**

a)not working b)has a regular job c)has an irregular job (occasional work) d) own work e)retired f)housewife

**8-Total income of your family?**

a)0-194 € b)195-247 € c)248-807 € d)808 € and up

**9-Do you have any chronic illness?**

a)No b)Hypertention c)Diabetes Mellitus d)Cardiac disease e)Hyperlipidemia f)Other

**10-Have you ever been treated for a psychiatric disorder?**

a)Yes (Diagnosis:……………………) b)No

**11-Are you still using drugs( constantly)?**

a)Yes (………………………….. ) b)No

**12-Do you have surgery before?**

a)Yes……………… b)No

**13-Have you ever been faced with a life-threatining situation?**

a)Yes b)No

**14-Have you ever been treated in the hospital?**

a)Yes (Diagnosis: length of stay: how many: ) b)No

**15-Have your close friend or relative ever been treated in the hospital?**

a)Yes b)No

**16-Do you have any plan or expectation for the future?**

A)Yes b)No c)Ever thought

**17-Have you ever thought of death wish?**

a)Yes ( go 18.question) b)No (go 19.question)

**18-If you ever thought death wish what was the reason of this?**

a)Economic b)Disease c)Family d)Other ...

**19-With which of the following choice do you explain not thinking of death before ?(the most effective one)**

a)Having plans for future

b)Not appropriate to your beliefs.

c)Family

d)Other …

**20-Have you ever got any relative or friend who is bedridden?**

a)Yes b)No

**21-Have you ever met with a patient about to die?**

a) Yes b)No

**22- If you have a serious illness (such as cancer) that can threaten your life in the future, would you like to have news from this situation?**

a)Yes b)No c)Not sure

**23-** **In the future, if your first-degree relatives (parents, siblings, spouse and children) are seriously ill or have a life threatening situation (such as cancer) in the future , would you like them to be informed of this?**

a)Yes b)No c)Not sure

**24-** **Should it be intervented (such as making a heart massage, connection to the respiratory device) when the person has a non treated or late diagnosed disease such as malignant cancer?**

a)Yes b)No c)Not sure

**25-If your relative is in the vegetative state because of an untreated disease, will it be appropriate to remove from the respiratory device?**

a)Yes b)No c)Not sure

**26-Do you believe that these interventions (heart massage, connection to the respiratory device) will have a positive effect on the patient's survival and / or life comfort in the last period of his / her life?**

a)Yes b)No c)Not sure

**27-Do you want your first-degree relatives (mother, father, siblings, spouse and child) be intervened in such a situation (in the last period, when the heart stops)?**

a)Yes b)No c)Not sure

**28-If you have an incurable disease, would you like to be intervened (heart massage, connection to the respiratory device) in the last period of your life?**

a)Yes b)No c)Not sure

**29-Do you want to make a decision about this situation when you are healthy and have intelligence abilities before this situation?**

a)Yes b)No

**30-Would you like to be intervened or not intervened according to this situation?**

a)Yes b)No

**31-Where do you want to be in the last period of your life or while you are dying?**

a)Hospital b)Own house c)Nursing home d)Other…

**32-Would you like to give these decisions to your family physician by writing them like a testament?**

a)Yes b)No

**33- Who do you want to talk to or get an idea of, when you make such a decision about yourself?**

a) Family

b)Relatives

c)Friends

d)Family doctor

e)The doctor who follows you

**34-Who do you want to decide about yourself in this situation (removal from the respiratory device, no heart massage)?**

a)Family

b)Friends

c)Family doctor

d)Relatives

e)The doctor who follows you

**35-Who do you want to talk to or get an idea of, when you make such a decision about your first-degree relatives?**

a)Family

b)Relatives

c)Friends

d)His/her family doctor

e)The doctor who follows him/her

**36-Which of the following would be the most effective in making this decision?**

a)İnformation about disease

b)Suggestions and discourses of people around you

c)Your beliefs and sociocultural values

d)Family doctor

**37-Do you consider yourself to be faithful?**

a)Yes b)No c)Partially
